# Genomic erosion in the assessment of species' extinction risk and recovery potential

**DOI:** 10.1093/jhered/esag011

**Published:** 2026-02-02

**Authors:** Cock van Oosterhout, Samuel A Speak, Thomas Birley, Lewis W G Hitchings, Chiara Bortoluzzi, Lawrence Percival-Alwyn, Lara Urban, Jim J Groombridge, Gernot Segelbacher, Hernán E Morales

**Affiliations:** School of Environmental Sciences, University of East Anglia, Norwich Research Park, Norwich, United Kingdom; School of Environmental Sciences, University of East Anglia, Norwich Research Park, Norwich, United Kingdom; Earlham Institute Norwich Research Park, Norwich NR4 7UZ, United Kingdom; School of Environmental Sciences, University of East Anglia, Norwich Research Park, Norwich, United Kingdom; School of Environmental Sciences, University of East Anglia, Norwich Research Park, Norwich, United Kingdom; SIB Swiss Institute of Bioinformatics Amphipôle Quartier UNIL-Sorge, Lausanne 1015, Switzerland; National Institute of Agricultural Botany (NIAB), Cambridge, United Kingdom; Institute for Food Safety and Hygiene University of Zurich, Winterthurerstrasse 272, Zürich 8057, Switzerland; Helmholtz Pioneer Campus, Helmholtz Zentrum München, Ingolstaedter Landstr. 1, Neuherberg 85764, Germany; Helmholtz AI, Helmholtz Zentrum München, Ingolstaedter Landstr. 1, Neuherberg 85764, Germany; Durrell Institute of Conservation and Ecology, School of Natural Sciences, University of Kent, Canterbury, United Kingdom; Wildlife Ecology and Management, University Freiburg, Freiburg im Breisgau, Germany; Globe Institute, University of Copenhagen, Copenhagen, Denmark; Department of Biology, Science for Life Laboratory, Lund University, Lund 223 62, Sweden

**Keywords:** genomic erosion, biodiversity, conservation, genomics, extinction

## Abstract

Many species are undergoing rapid population declines and environmental deterioration, leading to genomic erosion. Here we define genomic erosion as the loss of genetic diversity, accumulation of deleterious mutations, maladaptation, and introgression, all of which can undermine individual fitness and long-term population viability. Critically, this process continues even after demographic recovery due to a time-lagged impact of genetic drift, which is known as drift debt. Current conservation assessments, such as the International Union for Conservation of Nature Red List, focus on short-term extinction risk and do not capture the long-term consequences of genomic erosion. Likewise, the longer-term assessments of the International Union for Conservation of Nature Green Status may overestimate population recovery by failing to account for the enduring effects of genomic erosion. As genome sequencing becomes increasingly accessible, there is a growing opportunity to quantify genomic erosion and integrate it into conservation planning. Here, we use genomic simulations to illustrate how different genomic metrics are sensitive to the drift debt. We test how ancestral effective population size (N_e_) and bottleneck history influence the tempo and severity of genomic erosion. Furthermore, we demonstrate how these dynamics shape genetic load and additive genetic variation, which are key indicators of long-term evolutionary potential. Finally, we present a proof-of-concept for a Genomic Green Status framework that aligns genomic metrics with conservation impact assessments, laying the foundation for genomics-informed strategies to support species recovery.

## Introduction

Conservation biology has long been characterized as a “mission-oriented crisis discipline,” in which management actions must be taken rapidly, often with limited data and resources (Soulé 1985; McDonald-Madden et al. 2008; Wilson et al. 2011). Over the past decades, many species have been saved from extinction (Hoffmann et al. 2010; Bolam 2021). However, as the biodiversity crisis accelerates, human-induced environmental changes are causing rapid population declines across taxa (Watson 2019). Although demographic metrics such as census population size (N) and geographic range have guided most conservation policy to date, there is growing recognition that genetic factors critically influence species’ resilience, extinction risk, and capacity for recovery, particularly in the long-term (Frankham 2005; Exposito-Alonso et al. 2022; Forester et al. 2022; Wilder et al. 2023; van Oosterhout 2024; Shaw et al. 2025). This long-term perspective is of critical importance because current conservation and extinction-risk assessments, e.g. the International Union for Conservation of Nature (IUCN) Red List, focus on short-term dynamics over 3 years or 10 generations (whichever is longest) (IUCN 2004). Advances in genomic sequencing now allow us to analyze whole genomes to reconstruct recent changes in demography and evolutionary events, and to quantify genome-wide diversity and characterize functional and harmful genetic variation. These metrics can offer powerful insights into long-term population viability and adaptive potential (Soulé 1987; Charlesworth 2009; Lowe et al. 2017; Kardos 2021; Moran 2021; Forester et al. 2022; Willi 2022). Despite this potential, genomic data remain peripheral in conservation and extinction-risk assessments, and the explicit protection of genetic diversity continues to lag behind species- and ecosystem-level priorities (Laikre 2010; Willoughby 2015; Hoban 2023). This situation ultimately leaves a critical gap and fails to incorporate evolutionary processes into conservation planning (Hoffmann 2015; Cook and Sgrò 2019; Geue et al. 2025; Shaw et al. 2025).

### Defining genomic erosion

Genomic erosion is an umbrella term encompassing several genetic threats faced by many populations, including those arising from reduced effective population size as well as those resulting from maladaptive gene flow or introgression. Genomic erosion is often characterized by the progressive loss of genome-wide diversity resulting from historically reduced effective population sizes, such as those caused by population declines, bottlenecks, or fragmentation. Because it is shaped by ancestral demography, erosion can persist even in populations that are currently stable or recovering.

Genomic erosion can reduce additive genetic variation, the heritable component of trait variation that determines a population’s ability to evolve under selection. This can potentially lead to maladaptation, especially during rapid environmental change (Hoffmann et al. 2017). Genomic erosion can also be characterized by an increase in genetic load, defined as the reduction in average population fitness caused by the accumulation and expression of deleterious mutations (Bertorelle et al. 2022). Thus, genomic erosion undermines individual fitness, reduces long-term viability and adaptive potential, and ultimately elevates extinction risk. Importantly, genomic erosion often remains cryptic because after demographic decline, the population’s new mutation-drift equilibrium is reached only slowly, leading to a prolonged “drift debt” (Gilroy et al. 2017; Dussex et al. 2023b; Gargiulo et al. 2024; Pinto et al. 2024; Liu et al. 2025). In other words, drift debt means that the genetic consequences of a past bottleneck continue to unfold for many generations, even if the population’s numbers have already begun to recover, and even if 2 populations reach the same size but only one has passed through a bottleneck.

Finally, populations can also suffer genomic erosion if they are introgressed due to gene flow from another species or evolutionary significant unit (ESU). This type of genomic erosion potentially leads to a loss of unique genetic diversity, which is a process often referred to as genetic swamping (Todesco et al. 2016).

### Genomic erosion and extinction

Genomic erosion is a pervasive—but frequently overlooked—consequence of the many threats faced by wild populations, such as overexploitation, invasive species, emerging infectious diseases, hybridisation, pollution, and habitat and environmental change. These threats fundamentally alter the strength and direction of evolutionary forces. Specifically, the gene pool of threatened populations may experience more genetic drift and novel selection pressures (Mooney and Cleland 2001; Couvet 2002; Fogell 2021; Moran 2021). Moreover, altered patterns of gene flow and recombination can result in the introgression of the genome by heterospecific DNA (Rhymer and Simberloff 1996; Moran 2021), whilst environmental pollution can increase the (germline) mutation rate (Somers et al. 2002; Keith 2021). These genomic changes ca n reduce survival and reproduction, undermining overall population performance.

Although rarely the sole cause of extinction, genomic erosion interacts with demographic decline, habitat degradation, and other stressors to drive populations into genetic Allee effects (Luque et al. 2016), mutational meltdown (Lynch et al. 1995), insufficient adaptive evolutionary potential (Forester et al. 2022), and an extinction vortex (Fagan and Holmes 2006). Accordingly, genomic erosion often plays a critical role during the later stages of population decline, when the fate of a population or species is ultimately decided (Spielman et al. 2004). Moreover, the drift debt creates a time-lag in allele and genotype frequency changes, imposing a hidden genetic burden that may only become apparent several generations after the initial disturbance (Jackson et al. 2022; Pinto et al. 2024; Liu et al. 2025).

### Drift debt and genomic erosion

**Fig. 1:**
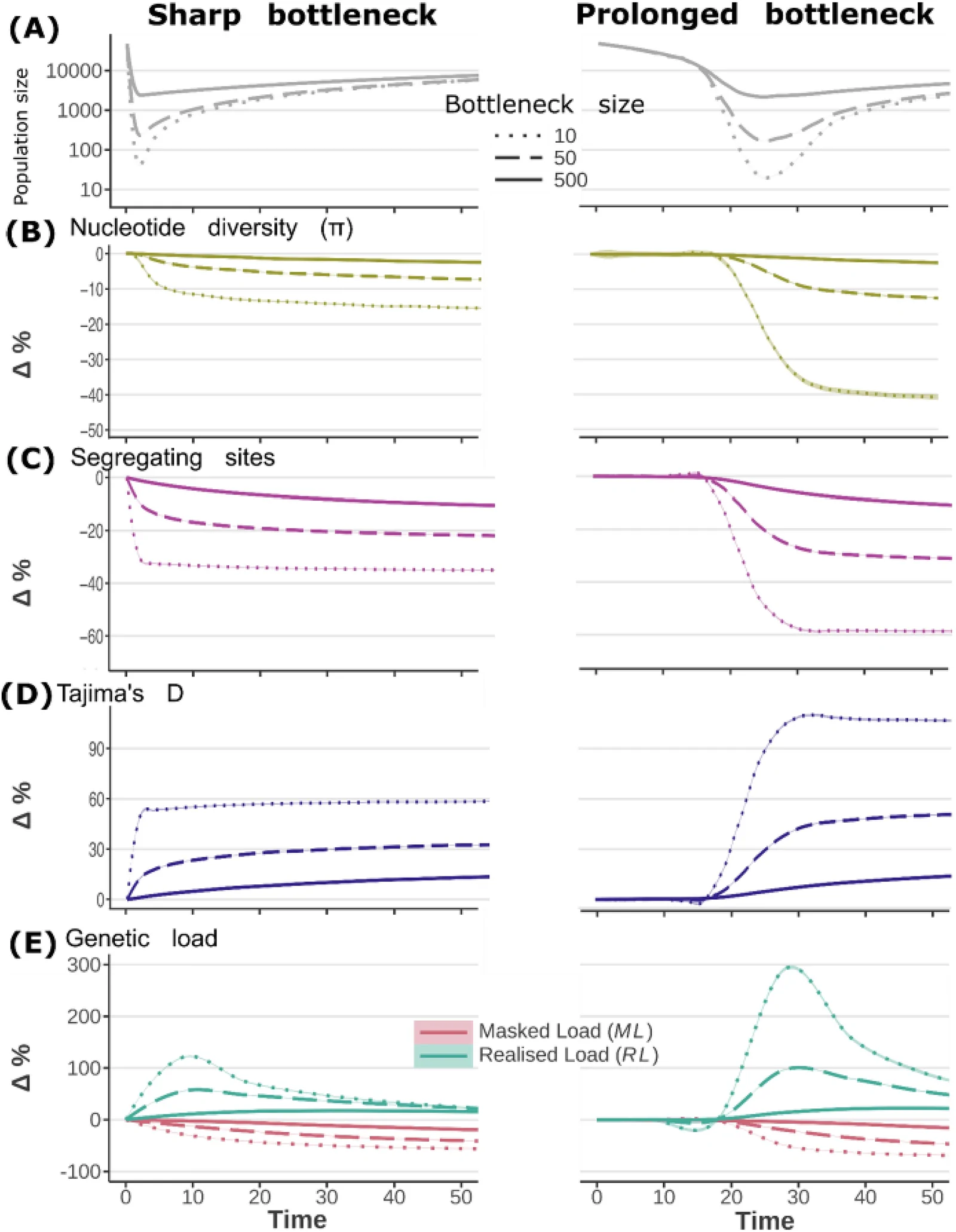
Genomic erosion metrics during population decline. Populations with an ancestral size of 10,000 breeding individuals were simulated in SLiM across contrasting bottleneck scenarios. The left panels depict fast and short bottlenecks (rapid decline over 1 generation and recovery after 2 generations), whereas the right panels show more gradual and prolonged bottlenecks (decline over 20 generations, recovery after 10 generations). (A) Demographic trajectories for 3 bottleneck intensities (Ne = 10, 50, 500). Panels (B–E) illustrate the temporal dynamics of 5 genomic metrics: (B) nucleotide diversity (π), (C) segregating sites (S), (D) Tajima’s D, and (E) genetic load components (RL) and masked load (ML)). These results highlight the contrasting temporal responses of genomic indicators to demographic change and the drift debt, revealing complementary insights into both immediate and delayed consequences of genomic erosion.

To visualize how this time-lag unfolds across different genomic features, we used forward-in-time simulations to illustrate the demographic and genetic consequences of drift debt under a range of bottleneck and population recovery scenarios (Fig. 1A). The timing and magnitude of responses varied across genetic metrics and bottleneck intensities. Among diversity statistics, nucleotide diversity (π) responded slowly because genetic drift continued to erode diversity for several generations after population size recovered, resulting in a pronounced drift debt (Fig. 1B). In contrast, the number of segregating sites (*S*) responded more rapidly, reflecting the swift loss of rare alleles, and stabilized shortly after recovery (Fig. 1C). As a consequence of this shift in the allele frequency spectrum, Tajima’s *D* became strongly positive during early recovery (Fig. 1D). Genetic load metrics showed similarly distinct temporal dynamics: realized load (RL) rose sharply after the crash, especially during the first 10 to 20 generations when inbreeding depression risk is highest (Fig. 1E), whereas masked load declined more gradually due to purging and conversion into RL (Fig. 1E). Together, these results reveal that different metrics capture distinct phases of genomic erosion, with genetic diversity loss and elevated RL persisting long after demographic recovery. This underscores the value of combining complementary genomic indicators, and ideally temporal genomic data, to assess both immediate threats to viability and long-term adaptive capacity.

The time-lag of genetic diversity loss is particularly problematic for conservation assessments because populations may initially appear genetically healthy right after population decline. Crucially, previously bottlenecked populations that show partial demographic recovery are often downlisted in the IUCN Red List. Yet, these populations may continue to lose genetic diversity due to drift debt, thereby increasing their extinction risk (Jackson et al. 2022; Fontsere et al. 2024). Additionally, some conservation actions can exacerbate genomic erosion, e.g by relaxing selection pressures through supplementary feeding in the wild or captive breeding in zoos (Araki et al. 2007; Frankham 2008; Robinson et al. 2023). Thus, even after immediate threats are mitigated, genomic erosion can persist as a long-term constraint on recovery and viability, potentially causing the Red List assessment to underestimate extinction risk.

**Box: the central role of N_e_ in extinction risk**

Conservation assessments have traditionally been focused on census population size (N) and geographic range, yet effective population size (N_e_) ultimately governs the balance between mutation, drift, and selection. As such, N_e_ shapes both the retention of adaptive variation and the accumulation of deleterious alleles (Frankham 2021; Laikre 2021; Waples 2025). Recently, effective population size (N_e_) has been proposed as a genetic diversity indicator for inclusion in the global biodiversity framework of the Convention on Biological Diversity (CBD), and as one of the genetic Essential Biodiversity Variables (EBVs) within the EBV class Genetic composition developed by the Group on Earth Observations Biodiversity Observation Network (GEO BON). However, many challenges remain in estimating N_e_ (Ryman et al. 2019; Fedorca et al. 2024). Confusingly, the N_e_ is an umbrella term that reflects the impact of genetic drift and inbreeding on different population genetic statistics (Waples 2025). In practice, this means that populations can have multiple, sometimes markedly different, N_e_ values depending on how this is estimated, such as the inbreeding N_e_, variance N_e_, and coalescence N_e_, which have been comprehensively reviewed in (Waples 2025). This variety in N_e_ estimators can lead to misguided interpretations for conservation. Moreover, N_e_ estimators have different temporal resolutions from the ancestral N_e_ (thousands of generations ago), the recent N_e_ (one to hundreds of generations ago), and the current N_e_ (Nadachowska-Brzyska et al. 2022).

Ancestral N_e_: When N_e_ estimation is based on nucleotide diversity or the coalescence of alleles, it is largely shaped by the genetic effective size of the ancestral population many thousands of generations in the past. This ancestral N_e_ relates to the equilibrium between the input of genetic variation by mutations and loss of this diversity due to genetic drift (Θ = 4N_e_μ). Software such as Pairwise Sequentially Markovian Coalescent (PSMC), Multiple Sequentially Markovian Coalescent (MSMC), and Bayesian Skyline Plots (BSP) are commonly used to reconstruct historical demography based on the coalescence of alleles. However, high recombination rates (relative to mutation rates) can make N_e_ inference unreliable (Bortoluzzi et al. 2023). Furthermore, recent population size declines reduce genetic diversity, but the coalescence of alleles and loss of nucleotide diversity are slow processes that are markedly affected by the drift debt (Fig. 1). Consequently, knowledge about ancestral N_e_ alone is of limited relevance for present-day extinction risk assessment of threatened species without proper context (see below).

Recent N_e_: This is the trend of Ne in the recent past (e.g. <100 generations ago). The linkage-disequilibrium (LD) N_e_ estimate responds more quickly to changes in population size as they reflect the evolutionary balance between recombination and inbreeding that is shaped by recent changes in demography over the past few hundred generations. Recombination reduces LD, whereas inbreeding (and hence, small N_e_) increases LD. Software such as GONE and SNeP can be used to infer this linkage-based estimate of N_e_, which can capture recent demographic events such as bottlenecks and founder events. Given that issues relating to inbreeding depression and the spike in RL play out across this relatively recent timescale (Fig. 1), this makes the recent N_e_ more directly relevant to conservation assessments. It is worth noting that substructure and gene flow can confound demographic reconstruction from LD (Novo et al. 2023).

Contemporary N_e_: This represents point (current) or very recent estimates of Ne. The most immediate estimate of N_e_ is based on loss in heterozygosity across one (or multiple) generations. For conservation genetic purposes, the contemporary N_e_ estimate is particularly useful because it reflects the status of diversity loss in the current population relative to a previous sample. Unfortunately, it requires temporal genomic samples from 2 (or more) generations, which has thus far limited its application. Moreover, admixture between previously isolated populations or distinct ESUs can artificially inflate contemporary N_e_ estimates, which emphasizes the importance of assessing the loss of diversity within ESUs (Geue et al. 2025).

Increasingly, conservation genetic studies report the N_e_/N_c_ ratio (Waples 2024). This ratio is inflated in many threatened species, especially in recently bottlenecked populations (Wilder et al. 2023; Wang et al. 2025). However, it is critically important to know how the N_e_ was estimated. In species that experienced a gradual decline in population size, the N_e_/N_c_ ratio based on ancestral N_e_ estimators is likely to be significantly inflated due to the drift debt. Afterall, nucleotide diversity and the coalescence of alleles change only slowly during population size decline. Hence, the ancestral N_e_ lags behind the census population size (N_c_), inflating the N_e_/N_c_ ratio (Wilder et al. 2023; Waples 2024; Wang et al. 2025). In contrast, when using a recent or current N_e_ estimate, the N_e_/N_c_ ratio is likely to be much less inflated by the drift debt.

### Genetic load and its role in extinction risk

Populations with large effective population sizes (N_e_) are expected to accumulate a substantial genetic load of partially recessive deleterious mutations at low frequency. These variants comprise the masked load, as their fitness effects remain largely hidden from selection while present in heterozygous form (Bertorelle et al. 2022). By definition, the masked load does not reduce mean fitness. However, when population size declines and inbreeding increases, homozygosity rises, converting masked load into RL (García-Dorado 2012; Hedrick and Garcia-Dorado 2016; Dussex et al. 2023b), which exposes deleterious effects and leads to inbreeding depression (Hedrick and Garcia-Dorado 2016; Smeds and Ellegren 2022). Furthermore, increased genetic drift in declining populations can elevate the frequency of harmful genetic variants, inflating homozygosity and RL even in the absence of close inbreeding (Pinto et al. 2024).

**Fig. 2:**
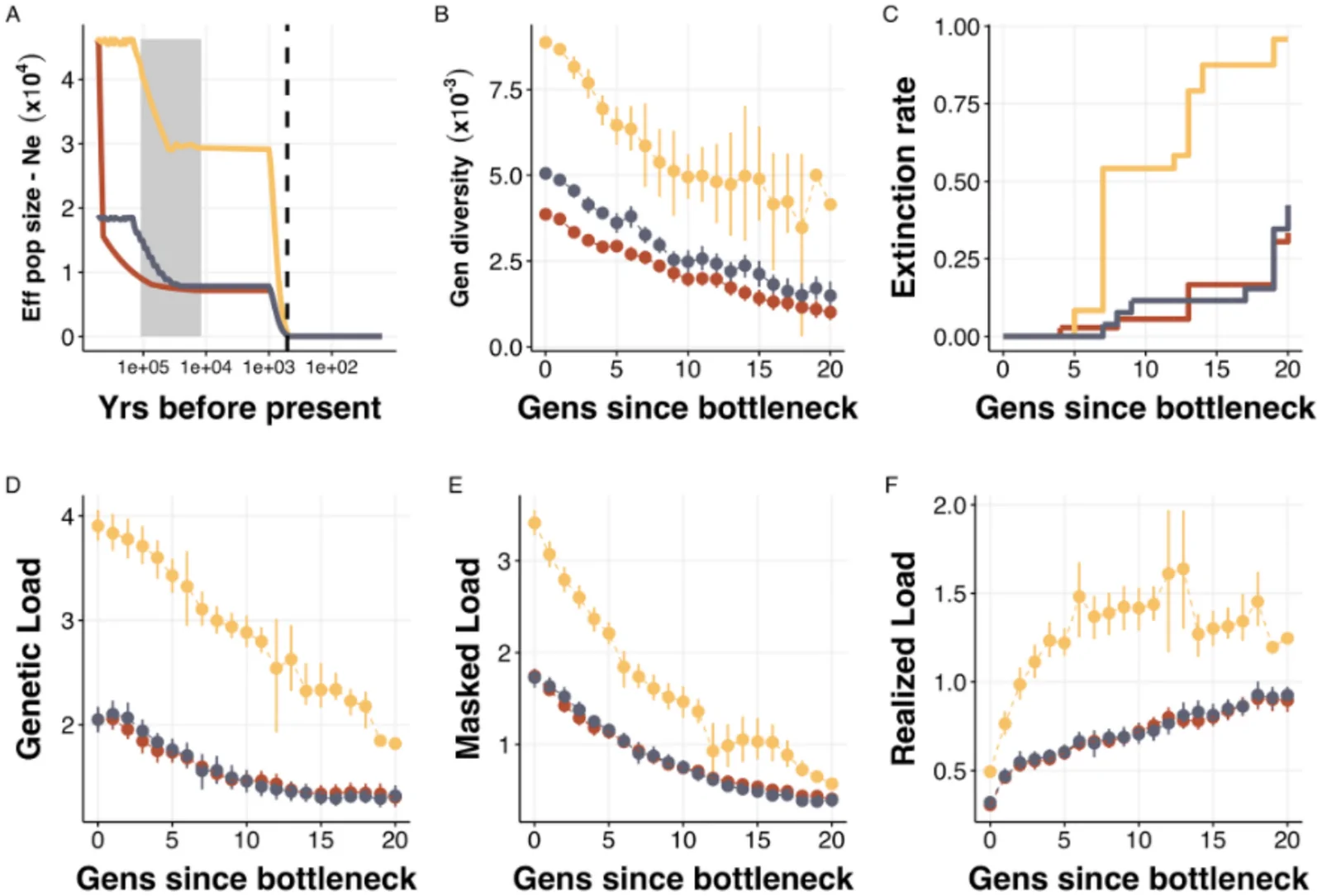
The effects of ancestral population size on genetic load dynamics. (A) Populations with distinctly different ancestral demographic trajectories experienced a severe population bottleneck (Ne = 10). Gray shading represents the last glacial period, 110,000 to 12,000 years ago. Dotted line represents the beginning of the Anthropocene in the year 1610. (B) Populations with historically large ancestral sizes show the highest nucleotide variation, but panel (C) shows that these populations also have the highest extinction rate after a bottleneck. (D) This is because the genetic load is highest in the populations with historically large ancestral sizes. (E) Historically, when the population was still large, the genetic load was not expressed, and this part of the genetic load is known as the masked load. (F) However, population size decline results in inbreeding, during which the masked load is converted into realized load (RL).

Accurate extinction risk assessment, therefore, requires reconstructing the demographic trajectory of N_e_ over time (Fig. 2A). Populations with large ancestral N_e_ accumulate more deleterious alleles as masked load and are at greater risk of severe inbreeding depression following demographic collapse (Grossen et al. 2020; Bertorelle et al. 2022; Kleinman-Ruiz 2022; Femerling et al. 2023; Dussex et al. 2023b). Forward-in-time simulations illustrate that ancestrally large populations harbor more nucleotide diversity (Fig. 2B) and higher masked load (Fig. 2E). After undergoing an equivalent severe bottleneck (to N_e_ = 10), these populations convert substantially more masked load into RL (Fig. 2F), resulting in markedly elevated extinction rates (Fig. 2C) compared to populations with smaller ancestral N_e_. Thus, large ancestral N_e_ can be a red flag for declining species, including zoo populations derived from a small number of founders. These insights are consistent with emerging empirical approaches such as the ID(risk) statistic (Kyriazis et al. 2025), which combines long ROH as evidence of recent inbreeding with heterozygosity in non-ROH regions as a proxy for masked deleterious variation to quantify the risk of inbreeding depression.

In addition to simulations, genomic data can be used to reconstruct historical demography and estimate genetic load across the genome. Advances in genome annotation and functional prediction (e.g. tools like CADD and GERP) allow estimation of deleterious variant burden (Kircher et al. 2014; Bertorelle et al. 2022; Speak et al. 2024). Predictions validated in model species can be transferred to threatened taxa (Fontsere et al. 2024; Speak et al. 2024; Wang et al. 2025), and emerging deep learning models further improve prediction accuracy (Frazer et al. 2021). Comparative genomic analyses confirm that genetic load scales with ancestral N_e_, and that populations with recent declines often suffer from elevated RL (Wang et al. 2025). Temporal genomic datasets are especially valuable, enabling direct observation of masked-to-RL conversion and providing a dynamic framework for assessing the impact of genomic erosion over time (Van Der Valk et al. 2019; Dussex 2021; Femerling et al. 2023; Dussex et al. 2023a; Bortoluzzi et al. 2024; Cavill et al. 2024; Fontsere et al. 2024).

### Genomic erosion limits current and future adaptation

**Fig. 3:**
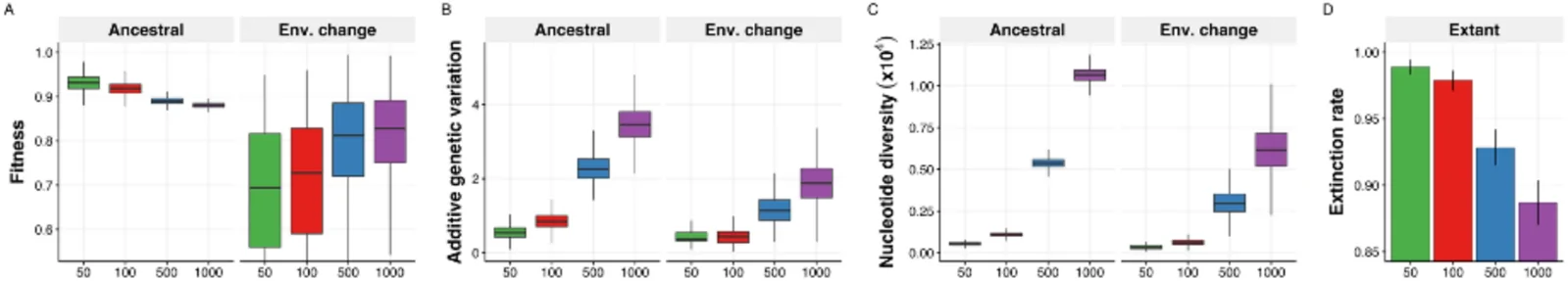
The effects of ancestral population size on adaptation. Computer simulations in SLiM of populations with different ancestral effective population sizes (Ne = 50, Ne = 100, Ne = 500, Ne = 1,000) with a trait adapted to an environmental optimum. The populations experience a severe population bottleneck (*Ne* = 10) that reduces genetic diversity. Five generations after this bottleneck, the environment changes, resulting in a shift of the optimum trait value. Here we show the distribution of values across 5 generations before the population bottleneck (ancestral) and 5 generations following the optimum shift (env. Change). (A) Larger ancestral populations have a slightly lower fitness because they possess more additive genetic variance (V_A_), conferring them more phenotypic variation around the environmental optimum, which constitutes a genetic load of conditionally deleterious mutations. (B-C) Larger ancestral populations also possess more additive genetic variation and genome-wide genetic diversity. However, after environmental change, the higher diversity in larger ancestral populations allows them to adapt to the new environmental optimum. Consequently, larger ancestral populations have a higher fitness after the optimum shift (A) and a lower extinction rate (D). V_A_ is positively correlated to neutral genetic diversity, highlighting the value of high genetic diversity to preserve adaptive potential. For simplicity, we simulated a single additive polygenic trait without environmental variance to illustrate the reduction of V_A_. Parameters such as dominance, epistasis, and the genetic architecture of the trait might temporarily increase V_A_ after a bottleneck (Goodnight 1988; Willis and Orr 1993; Barton and Turelli 2004). However, over time, genetic drift is expected to lead to a reduced adaptive response under most conditions.

Polymorphisms at quantitative trait loci (QTL) can be either deleterious or beneficial depending on the genetic background and environmental conditions (Charlesworth 2013a, 2013b; Kardos 2021). Most outbred populations are adapted to their environmental optimum as additive genetic variation at QTL is maintained by stabilizing selection acting on the trait (Charlesworth 2013a). Thus, genomic erosion could lead to maladaptation by removing additive genetic variation, and the outcome of this process depends on the ancestral N_e_ (Fig. 3). Perhaps counterintuitively, populations with a large ancestral N_e_ have on average a lower fitness from traits under stabilizing selection (Fig. 3) because larger populations are closer to the trait optimum so any new mutation will be (on average) more deleterious (Charlesworth 2013a). However, the amount of additive genetic variation segregating in the ancestral population also underpins their adaptive evolutionary response during environmental change. Hence, populations with large ancestral N_e_ are better able to respond to environmental change, and theoretically, they are expected to have a lower extinction risk than small populations (Fig. 3). On the other hand, as previously shown, during population decline ancestrally large populations are more prone to inbreeding depression due to a higher masked genetic load (Fig. 3). Thus, high ancestral N_e_ is a double-edged sword: it promotes historical diversity but allows deleterious variants to persist at low frequency, only to manifest as inbreeding depression and elevated extinction risk under collapse. Moreover, maladaptation may also arise from gene–environment mismatches, particularly under climate change, where formerly adaptive traits may become deleterious in novel environmental conditions. This highlights the importance of assessing the multifarious threats of genomic erosion using computer models of “digital twins” that incorporate the effects of the demographic history, different types of genetic variation, selection regimes, and realistic rates of environmental change (Forester et al. 2022; Jackson et al. 2022; Robinson et al. 2022; Nigenda-Morales et al. 2023; Pinto et al. 2024).

Similarly, the loss of immunogenetic diversity constitutes a key component of genomic erosion. The major histocompatibility complex (MHC) and toll-like receptors (TLRs) are among the most studied immune loci, and their variation is typically subject to balancing selection that maintains high allelic diversity within populations (van Oosterhout 2009; Spurgin and Richardson 2010; Gilroy et al. 2017). However, bottlenecked populations can lose immunogenetic diversity, which makes them more susceptible to disease outbreaks (Grueber et al. 2012; Morris et al. 2015; Dalton et al. 2016; Fogell 2021; Silver et al. 2025). Erosion of immunogenetic diversity does not necessarily proceed at a similar rate as neutral diversity (Gilroy et al. 2017; Lighten et al. 2017), which highlights the need to monitor functional loci alongside genome-wide markers. Maintaining immunogenetic diversity is critical for managing disease risk in small populations, guiding translocations, and identifying targets for gene editing aimed at restoring functional variation (Morris et al. 2015; Silver et al. 2025; van Oosterhout et al. 2025).

### Genomic modeling to forecast extinction and recovery

To assess the long-term risks posed by genomic erosion, conservation efforts must integrate genomic data analysis with computer modeling (Kardos 2021; Kyriazis et al. 2023; Mathur et al. 2023; Dussex et al. 2023b). For decades, conservation scientists have relied on population viability analyses (PVA) to assess threats and inform conservation actions (Lacy 2019). Although traditional PVA models can incorporate genetic data to assess the effects of inbreeding on population viability, they were not designed to capture genome-wide patterns of erosion. The value of evolutionary theory, computer modeling, and genomics is increasingly recognized in conservation, with the latter 2 fields advancing especially rapidly (Frankham et al. 2019; Funk et al. 2019; Hohenlohe et al. 2021; Segelbacher 2022; Willi 2022; Shaw et al. 2025). A new generation of evolutionary genomics models enables the construction of complex, genome-scale simulations that integrate demographic, ecological, and evolutionary dynamics within a unified framework (Guillaume and Rougemont 2006; Haller and Messer 2019, 2023; Terasaki Hart et al. 2021). Figures 1 to 3 show that such simulations can provide baseline expectations for how different bottleneck severities and recovery trajectories shape genomic erosion. This modeling framework can be extended to incorporate species-specific traits and ecological contexts for more realistic predictions.

Genomic data provide the foundation for these models. Demographic inferences, mutation rates (e.g. from parent-offspring trios (Bergeron et al. 2023)), and recombination landscapes (Peñalba and Wolf 2020) can be used to parameterise realistic genome architectures. These models can also incorporate species-specific traits such as reproductive strategy, dispersal, and longevity, and be made spatially explicit to assess the impact of metapopulation dynamics or habitat fragmentation (Pinto et al. 2024). Crucially, the simulated outcomes of such “digital twins” can be compared directly to empirical genomic datasets for validation, enabling predictions under different environmental and management scenarios.

Forward-in-time simulations are increasingly applied to forecast the impacts of climate change, land-use change, loss of connectivity, adaptive potential, and genetic rescue (Matz et al. 2018; Brauer and Beheregaray 2020; Dussex 2021; Hansson et al. 2021; Kyriazis et al. 2021; Stoffel et al. 2021a; Jackson et al. 2022; Magliolo 2022; Beichman et al. 2023; Femerling et al. 2023; Kyriazis 2023; Kyriazis et al. 2023; Dussex et al. 2023b; Al Hikmani et al. 2024; Cavill et al. 2024; Pinto et al. 2024; Liu et al. 2025). Yet challenges remain: accurate model parameterisation requires genomic and ecological data that are still lacking for many species, and comparative frameworks are only now emerging. Increasing availability of temporal genomic datasets opens a powerful opportunity to validate forward-in-time simulations by directly comparing simulated genomic trajectories to observed changes over time. Such validation allows researchers to assess whether simulations accurately capture the pace and magnitude of genetic erosion following demographic collapse or recovery, including shifts in heterozygosity, allele frequency spectra, or RL. Incorporating fitness data alongside genomic metrics further strengthens this framework, enabling the evaluation of how well predicted genetic load or inbreeding depression translates into fitness declines in real populations. As the volume and resolution of genomic time series increase, so too does the ability to calibrate models not only on past dynamics but to project genetic outcomes under alternative management actions or future environmental change. This approach transforms simulations from abstract scenarios into empirical, testable tools for forecasting extinction risk, recovery potential, and the long-term consequences of conservation interventions.

### Integrating genetic risk into extinction assessments

There is mixed evidence as to whether Red List categories consistently reflect underlying levels of genetic diversity. Some studies show that threatened species tend to have lower genetic diversity than non-threatened species, but this pattern is not universal and appears to vary depending on the taxa, genetic markers, and metrics used (Willoughby 2015; Brüniche-Olsen et al. 2021; Canteri 2021; Schmidt et al. 2023; Jeon et al. 2024; McLaughlin et al. 2025; Wang et al. 2025). Recent efforts have called for harmonized workflows and core genomic metrics, highlighting the importance of consistency in data generation and the selection of biologically meaningful, conservation-relevant indicators (Buzan et al. 2024; Jeon et al. 2024; McLaughlin et al. 2025).

Inconsistencies have sparked debate over whether the Red List can effectively protect intraspecific genetic diversity. Conversely, others have questioned whether, given this poor association, genetic data can be used to assess extinction risk (Canteri 2021; Schmidt et al. 2023; McLaughlin et al. 2025). We argue that both sets of data, ecological and demographic data collated in the Red List, and genetic or genomic data, are complementary, and that cover each other’s blindspots (van Oosterhout 2024). Neither the Red List nor the Green Status of Species assesses the impacts of genomic erosion. We stress that including genomic data in the Red List is critical because the loss of adaptive potential in combination with rapid environmental change poses unprecedented threats to wildlife. We therefore must assess the long-term viability of populations and species against the backdrop of environmental change, which requires analyses of genomic data, forward-in-time computer simulations, and Deep Learning models to decipher signals associated with elevated risk of extinction (van Oosterhout 2024).

### Genomic green status: Integrating genomic metrics into recovery assessments

The IUCN recently developed the Green Status of Species, which is a framework for measuring species recovery and conservation impact (Akçakaya et al. 2018; Grace et al. 2021). The assessment calculates a Green Score that quantifies the viability, functionality and representation of a species, and this metric ranges between 0% (extinct) to 100% (fully recovered). The Green Scores are measured at 4 different timepoints (ancestral, current, next 10 years, and next 100 years), and differences in the Green Scores between those timepoints are used to estimate 4 conservation impact metrics. *Conservation Legacy* estimates the impact of past conservation on the population or species, comparing it to a counterfactual scenario without any conservation actions. *Conservation Dependence* assesses how much worse the species is likely to be after 10 years without any conservation. Conversely, *Conservation Gain* measures the potential improvement of the species after 10 years with conservation actions. Finally, *Recovery Potential* aims to assess the long-term improvement that could be accomplished during 100 years with continued conservation.

Given its longer timeframe, the Green Status of species could incorporate the dynamics of genomic erosion, consistent with the time-lag and long-term effects of the drift debt. Importantly, it also offers a clear conceptual framework for the integration of genomic data because its 4 conservation impact metrics are dimensionless units with scalar property. In other words, the percentages of metrics relating to different aspects of the species (e.g. genome-wide diversity, genetic load, individual fitness, etc.) can be directly compared across time and species. Importantly, the conservation metrics are proportional statistics that measure the change expected under a hypothetical scenario relative to the status of the species at present. Therefore, genomic indicators can be directly aligned with the Green Status metrics of *Recovery Potential* and *Conservation Gain*, offering a means to quantify the genomic dimension of long-term species extinction risk and recovery potential.

To demonstrate how genomic data can be integrated into the IUCN Green Status framework, we developed a simulation-based framework to adapt the current implementation focusing on demographic change to instead quantify genomic recovery using indicators of genetic load and genome-wide diversity. We use the pink pigeon (*Nesoenas mayeri*) as an example of a species that underwent a severe bottleneck (N_e_~ 12 during 1990’s) followed by a demographic recovery through intensive conservation (currently N_e_~ 488 adult birds). However, due to the drift debt, its long-term survival is threatened by genomic erosion (Jackson et al. 2022). The simulation model captures the species’ historical demography and recent management interventions, including genetic and demographic rescue from a captive population founded by 12 individuals in the 1970s, as implemented in Jackson et al. (2022).

**Fig. 4:**
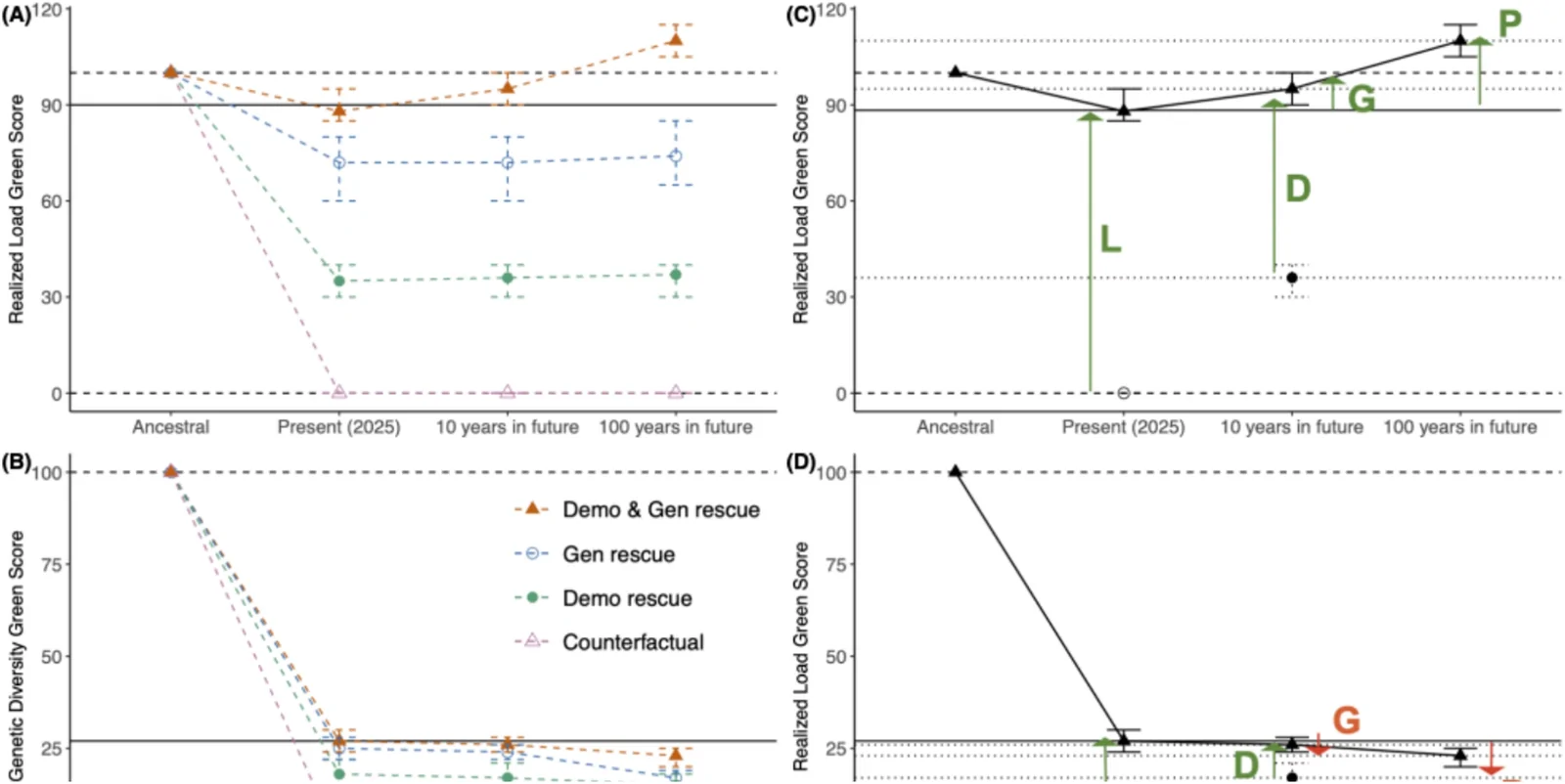
Genomic green scores for RL and genetic diversity across 4 simulated conservation scenarios. The left panels show the change in (A) RL and (B) genome-wide diversity over time for 4 conservation scenarios: (1) no conservation (counterfactual), (2) demographic rescue only, (3) genetic rescue only, and (4) combined demographic + genetic rescue. The right panels show the genomic green scores for (C) RL and (D) genome-wide diversity for the best-performing scenario of combined demographic + genetic rescue. Arrows show the change in SRS across time for conservation legacy (L), conservation dependence (D), conservation gain (G), and recovery potential (P). For genome-wide diversity, conservation gain (G), and recovery potential (P) are negative because of continued drift debt, which may jeopardize future adaptive potential. In contrast, the RL green scores continue to improve due to continued purging of deleterious mutations.

We modeled 4 scenarios: (1) no conservation (counterfactual), (2) demographic rescue only, (3) genetic rescue only, and (4) combined demographic + genetic rescue. For each, we tracked realized genetic load, nucleotide diversity, and extinction rates across time (Fig.  4). We adapted the Green Scores to calculate Species Recovery Scores (SRS) based on RL and genome-wide diversity. For genomic metrics, the interpretation of SRS depends on the directionality of the indicator. For RL, higher SRS values indicate a larger deviation from the ancestral (low-load) state and therefore reflect poorer genetic condition, whereas for nucleotide diversity, higher SRS values reflect closer value to ancestral diversity. For the RL, the current SRS is 88.3%, meaning the population retains ~ 88% of the excess harmful variation accumulated since the bottleneck, and therefore remains substantially worse than the ancestral (low-load) state. However, when compared to the counterfactual scenario, the *Conservation Legacy* shows that conservation interventions likely saved the species from extinction. *Conservation Dependence* is high (52.6%), showing that ongoing genetic supplementation remains crucial. *Recovery Potential* is also substantial (23.5%), as continued management is expected to reduce RL below ancestral levels via purging. In contrast, the Green Status for nucleotide diversity shows a lower SRS (25.4%), with modest Conservation Dependency (6.0%) and negative Recovery Potential (−8.7%), indicating that diversity loss is largely irreversible under current conservation scenarios.

By comparison, according to its Green Status assessment from 2021 (https://www.iucnredlist.org/species/22690392/179390191), the SRS for the Pink Pigeon is 17% (categorized as “Critically Depleted”), primarily reflecting extensive forest loss across its original range. It demonstrates a *High Conservation Legacy* between 14% and 17%—meaning that without prior conservation efforts, the species would very likely be extinct today. Its *Conservation Dependence* is *Low* between 5% and 8%, which means that if conservation efforts ceased, ecological functionality would deteriorate over a decade. Projected *Conservation Gain* over 10 years is also *Low* and between 5% and 17%, indicating that the species has limited potential recovery. However, its long-term *Recovery Potential* over a 100-year timeframe is significantly higher, scoring between 15% and 36%, implying that restoration of sufficient habitat could allow ecological functionality in many areas. Taken together, the ecological and genomics Green Status assessments are complementary. However, the genomic Green Status adds an important dimension. First, by evaluating the genetic health of the species, the genomics assessment highlights the urgent need for genetic rescue. Second, the comparatively high long-term Recovery Potential (P) in the ecological Green Status may be overly optimistic, as it does not account for genomic erosion and drift debt, which can substantially constrain recovery even when habitat rebounds (Table 1).

**Table 1:** Comparison of the ecological green status assessment and 2 genomic assessments based on RL and genome-wide diversity. Values are shown for all 5 green status components. Ecological values are taken from the 2021 IUCN green status assessment for the pink pigeon, whereas genomic scores are derived from our simulation-based framework. The divergence between ecological and genomic values demonstrates that genomic indicators capture dimensions of recovery and vulnerability from genomic erosion that ecological indicators alone do not reflect.

Metric	Ecological green status	Genomic green status RL	Genomic green status genome-wide diversity
SRS	17%	88.3%	25.4%
Conservation legacy (L)	14% to 17%	88.3%	25.4%
Conservation dependence (D)	5% to 8%	52.6%	6.0%
Conservation gain (G)	5% to 17%	59%	9.0%
Recovery potential (P)	15% to 36%	23.5%	−8.7%

### The future of genomics-informed conservation

The study of genomic erosion is a rapidly advancing field, yet it faces several key challenges. Recent genomic analyses have shed light on how population declines and recoveries affect the balance between purging and accumulation of deleterious mutations, potentially shaping long-term fitness and viability in small populations (Grossen et al. 2020; Dussex 2021; Humble 2022; Kleinman-Ruiz 2022; Riaño 2022; Smeds and Ellegren 2022; Femerling et al. 2023; Kyriazis 2023; Mathur et al. 2023; Dussex et al. 2023b; Fontsere et al. 2024). However, estimating additive genetic variation and directly linking genetic load with fitness effects remains difficult, especially in wild populations. Long-term monitoring programs, particularly those that incorporate fitness and genomic data across generations, offer one of the most promising avenues for quantifying adaptive potential and predicting extinction risk (Harrisson et al. 2019; Villemereuil 2019; Fogell 2021; Stoffel et al. 2021b; Bonnet et al. 2022; Jackson et al. 2022; Smeds and Ellegren 2022; Kardos et al. 2023; Hewett et al. 2024; Morales et al. 2024a; Morales et al. 2024b; Morales et al. 2024c).

Genomics-informed management is poised to become central to the conservation of both wild and captive populations. Zoo populations, often founded by very few individuals and exposed to relaxed selection in artificial environments, are especially vulnerable to genomic erosion. Genomic tools can help avoid unintended hybridization, limit the fixation of deleterious mutations, and detect adaptation to captivity. By monitoring allele frequency changes and minimizing artificial selection, genomic screening can reduce maladaptation and improve the success of reintroductions into the wild (Schulte-Hostedde and Mastromonaco 2015). Similarly, in genetic rescue programs, targeted genome-wide screening enables the identification of optimal populations or individuals that maximize diversity while minimizing the introduction of harmful mutations (Ralls et al. 2020; Kyriazis et al. 2021; Mathur et al. 2023; Speak et al. 2024). Several existing initiatives, such as the integration of genomic data into the Zoological Information Management System (ZIMS) and the development of large-scale biobanks, are already laying the groundwork for the systematic incorporation of genomic data into conservation practice (Schwartz et al. 2017; Pérez-Espona and CryoArks Consortium 2021; Mooney et al. 2023).

To translate genomic insights into conservation practice, efforts must focus on developing standardized frameworks for calculating and reporting genomic erosion across taxa, including historical baselines derived from museum samples (Díez-del-Molino et al. 2017; Buzan et al. 2024). Importantly, common metrics that capture genetic load, diversity, and adaptive potential should be defined to enable meaningful cross-species comparisons to guide conservation priorities (Jeon et al. 2024; Wang et al. 2025). The technical complexity of genomic analyses also requires harmonized pipelines and collaborative infrastructure, ensuring that conservation biologists, genomicists, bioinformaticians, and modelers can work together effectively.

Ultimately, a genomics-informed approach will allow conservation science to move from descriptive diagnostics to predictive frameworks. This shift will improve our ability to forecast extinction risk, measure conservation impact, and design recovery plans that secure the genetic health and evolutionary potential of species for generations to come.

## Methods

### Population bottleneck simulations

Simulations were performed in SLiM4 (Haller and Messer 2023) with a non-Wright-Fisher model adapted to non-overlapping generations and random mating for simplicity, where the number of simulated individuals corresponds to Ne. We simulated a genomic region modeled after chromosome 23 of the collared flycatcher genome (12.3 Mb) (Kawakami et al. 2014), incorporating realistic exon, intron, and intergenic region positions, as well as an underlying recombination map, thereby accurately representing linkage dynamics. We also simulated an exome architecture of 5 autosomes, each containing 1500 genes of 1500 bp with recombination rates of 1e-8 within genes and 1e-3 between genes. A global mutation rate of 1.5e-8 was used. Both neutral and deleterious mutations were simulated in ratios of 5:1 for introns, 1:2.31 for exons, and 1:0 for non-coding regions. Deleterious selection coefficients (s) were taken from a gamma distribution (mean = −0.05 and shape = 0.5) with a tail of 5% of lethal mutation and negative relationship between s and dominance coefficients (h), following (Kardos et al. 2021).

The population size was controlled by limiting the number of breeding individuals each generation, with each breeding pair producing 12 offspring. Populations all had an ancestral size limited to 10 000 breeding individuals. We explored different bottleneck decline speeds (1 and 20 generations), bottleneck durations (2 and 10 generations), and bottleneck sizes (10, 20, 50, 100, and 500 breeding individuals) with each combination of parameters being run for 100 replicates.

### Genetic load simulations

We used the same modeling approach as in (Dussex et al. 2023b). Briefly, simulations were performed in SLiM3 (Haller and Messer 2019) with a non-Wright-Fisher model adapted to non-overlapping generations and random mating for simplicity. The model simulated an exome of 3000 genes of 3.4Kb each with a recombination rate r = 1e-4 (no recombination within genes), and a per-base mutation rate m = 1.4e-8. Deleterious selection coefficients (s) were taken from a gamma distribution (mean = −0.05 and shape = 0.5) with a tail of 5% of lethal mutation and negative relationship between s and dominance coefficients (h), following Kardos et al. 2021. We ran 100 replicates per scenario.

### Additive genetic variation simulations

We used the same modeling approach as in (Femerling et al. 2023). Briefly, simulations were performed in SLiM3 (Haller and Messer 2019) with a non-Wright-Fisher model adapted to non-overlapping generations and random mating for simplicity. The model simulated an exome of 3000 genes of 3.4Kb each with a recombination rate r = 1e^−4^ (no recombination within genes), and a per-base mutation rate m = 1e^−7^. Fitness was determined based on the additive effect of genotype values (z) on a polygenic trait tracking an environmental optimum (opt) following (Falconer and Mackay 1996). Genotype values (z) were drawn from a uniform distribution from −0.5 to 0.5 and had a fixed additive effect (h = 0.5). The phenotype (P) of an individual was the sum of all homozygous and heterozygous effects. To calculate the fitness effect from the deviation of the phenotype (P) to the environmental optimum (opt) as w = (P − opt)^2^ and the additive genetic variation as V_A_ = Σ2p_i_q_i_z_i_^2^. We ran 100 replicates per scenario and counted the proportion of replicates that went extinct to obtain the extinction rate per scenario.

### Genomic green status simulations

We used simulated data from (Jackson et al. 2022). Briefly, simulations were performed in SLiM3 (Haller and Messer 2019) with a non-Wright-Fisher implementation, which considers overlapping generations, age-structure, and customizable offspring generation and migration patterns. During the simulation, each time step consists of 3 stages: reproduction, dispersal (between captive and wild populations, if any), and mortality. Absolute fitness (i.e. probability of survival) was regulated by the carrying capacity and the known age-based probability of mortality for pink pigeons. The model simulated an exome of 4000 genes of 3.4Kb each with a recombination rate r = 1e-4 (no recombination within genes), and a per-base mutation rate m = 7.5e-8. We modeled neutral genetic variation and a genetic load of ∼15 LEs as observed in the empirical data (see Jackson et al. 2022).

We simulated a demographic trajectory that captured the trend observed in the pink pigeon by controlling an overall carrying capacity informed by the inferred pre-1980s population size and recorded census data since 1980. The wild population began from an ancestral population size of 16,000 individuals, declined to ~10 birds by 1990, and subsequently recovered to ~400 individuals by the mid-2000s. The captive population used for genetic rescue was founded by 12 individuals in 1976 and increased to an average of ~ 120 birds. Genetic supplementation followed the empirically recorded release schedule, including the 47 birds translocated between 1994 and 1996, with additional releases continuing through 2019. We simulated 4 conservation scenarios across 40 replicate runs each: (1) Counterfactual (no recovery, no genetic supplementation); (2) Demographic rescue (population rebound without genetic rescue); (3) Genetic rescue (genetic supplementation without demographic increase); and (4) Demographic + genetic rescue, reflecting the actual conservation history of the species. Each replicate tracked nucleotide diversity (π), RL (sum of homozygous deleterious mutations weighted by s), and extinction over time.

Green Status metrics were calculated for both RL and nucleotide diversity across the 4 simulated scenarios. The present-day Green Score (SRS) was compared to hypothetical counterfactuals to calculate 4 conservation impact metrics: Conservation Legacy (L), Conservation Dependence (D), Conservation Gain (G), and Recovery Potential (R), following the IUCN Green Status framework. Metrics were derived by calculating proportional differences in Green Scores at different timepoints (ancestral, current, 10-year, and 100-year future) under contrasting conservation scenarios.

The Green Score of genetic diversity at time t is expressed relative to ancestral variation and calculated as π_t_/π_ancestral_ × 100%. Where π_t_ is the mean nucleotide diversity calculated over the entire population at time t, and π_ancestral_ the mean ancestral nucleotide diversity in the population. The Green Score of RL is expressed as the RL in the ancestral variation relative to the RL in the population at time t. It is calculated for the proportion of simulation runs that survived at time t (P_survived at time t_), giving extinct runs a score of zero. The Green Score of the RL is calculated as ({2 × RL_ancestral_}/{RL_t_ + RL_ancestral_}) × P_survived at time t_ × 100%. Where RL_ancestral_ and RL_t_ are the mean RL in the ancestral population and the population at time t, respectively. Note that to express the Green Score of RL in negative direction, in this equation, the nominator and numerator are switched relative to the Green Score of genetic diversity. Inbreeding and drift initially increase RL, resulting in a decline in the Green Score of RL. However, purging reduces the RL, causing its Green Score to improve relative to the ancestral population, resulting in a Green Score in excess of 100%.

## Data Availability

The code used to support the arguments in this perspective is openly available in https://github.com/hmoral/genomic_erosion_perspective.
